# Recurrent osteomyelitis with proliferative periostitis after segmental resection and reconstruction of the mandible: a case report

**DOI:** 10.1007/s10006-022-01051-4

**Published:** 2022-03-17

**Authors:** Michael Maurer, Josef Maximilian Gottsauner, Andreas Mamilos, Torsten E. Reichert, Tobias Ettl

**Affiliations:** 1 Department of Oral and Maxillofacial Surgery, University Hospital Regensburg, Franz-Josef-Strauss-Allee 11, 93053 Regensburg, Germany; 2 Institute of Pathology, University Hospital Regensburg, Franz-Josef-Strauss-Allee 11, 93053 Regensburg, Germany

**Keywords:** Osteomyelitis, Proliferative periostitis, Microvascular surgery, Mandible, Fibula

## Abstract

A 50-year-old patient presented with a two-year history of chronic osteomyelitis of the left mandibular body. It was treated by wide segmental resection of the left hemimandible and reconstruction with a free vascularized fibular graft. Six months after surgery, the patient returned with pain, swelling, and moth-like lesions in the transplant in combination with appositional bone formation surrounding the ossified fibular bone. Radiographic and histological examination led to the diagnosis of a recurrent osteomyelitis with proliferative periostitis affecting the resected and reconstructed mandible. Application of ibandronate led to a significant symptom decrease.

## Introduction

Chronic osteomyelitis with proliferative periostitis (COMPP) or periostitis ossificans describes a rare chronic osteomyelitis with associated periosteal new bone formation. The disease is traditionally known as Garré’s osteomyelitis. It usually occurs in the mandibular body of children and adolescent patients probably due to higher periosteal osteoblastic activity [1, 2]. Etiologically, low-virulent infections like dental caries, periapical lesions but also retained tooth germs, lead to apposition of immature vital bone layers outside the preexisting cortical layer [2, 3]. A radiopaque lamination is observed parallel to the surface of the cortical bone [2].

In the following, we present an atypical case of recurrent chronic osteomyelitis with an indistinct new periosteal bone formation after wide segmental mandibular resection and reconstruction with a vascularized fibula bone graft in an adult patient.

## Case report

**Fig. 1:**
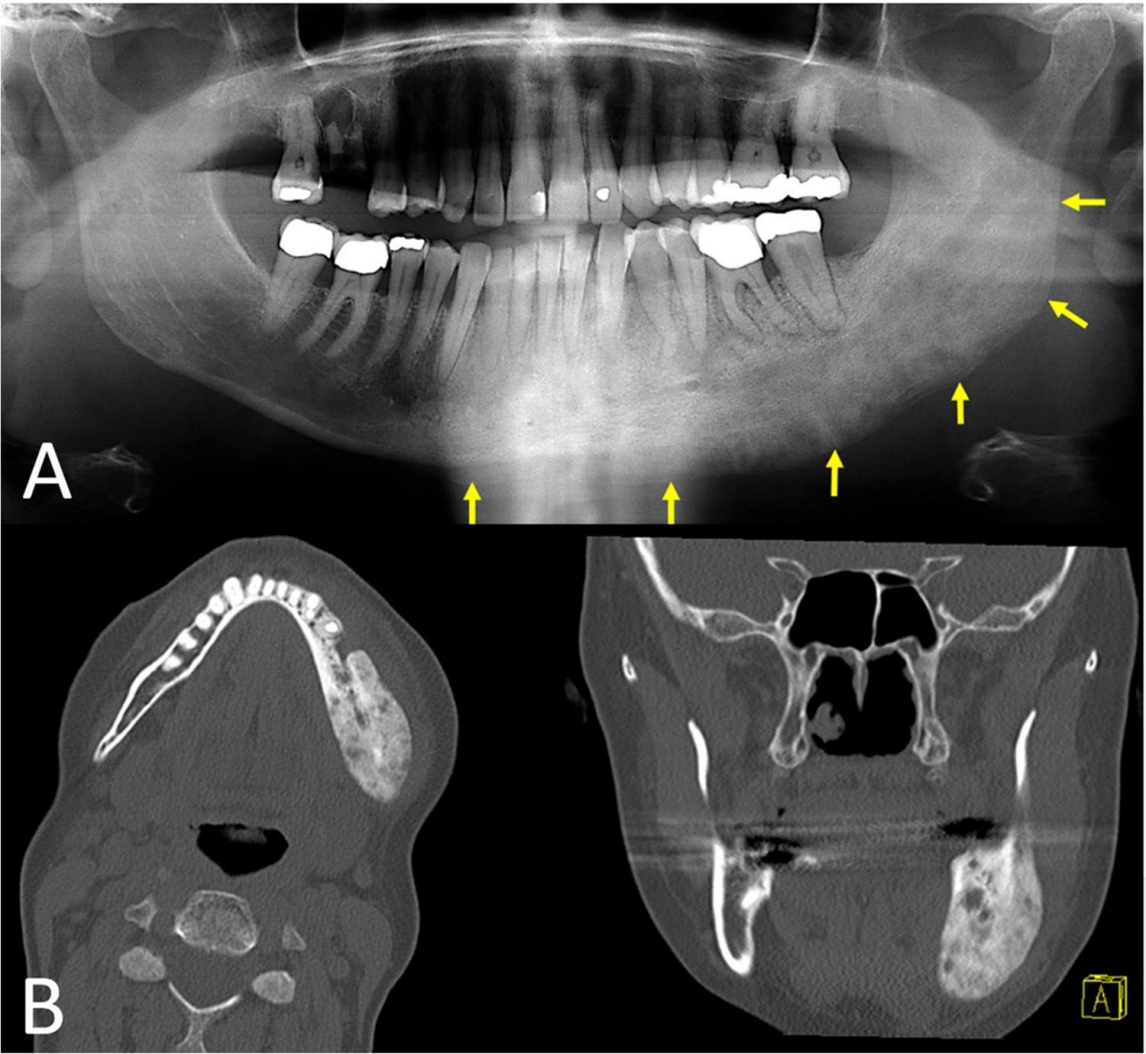
Initial situation in panoramic radiograph (**A**) and CT scan (**B**). Distension and deformity of the left hemimandible with both sclerosis of the cancellous bone and osteolytic lesions extending from the right premolar region to the posterior border of the ramus (arrows)

**Fig. 2:**
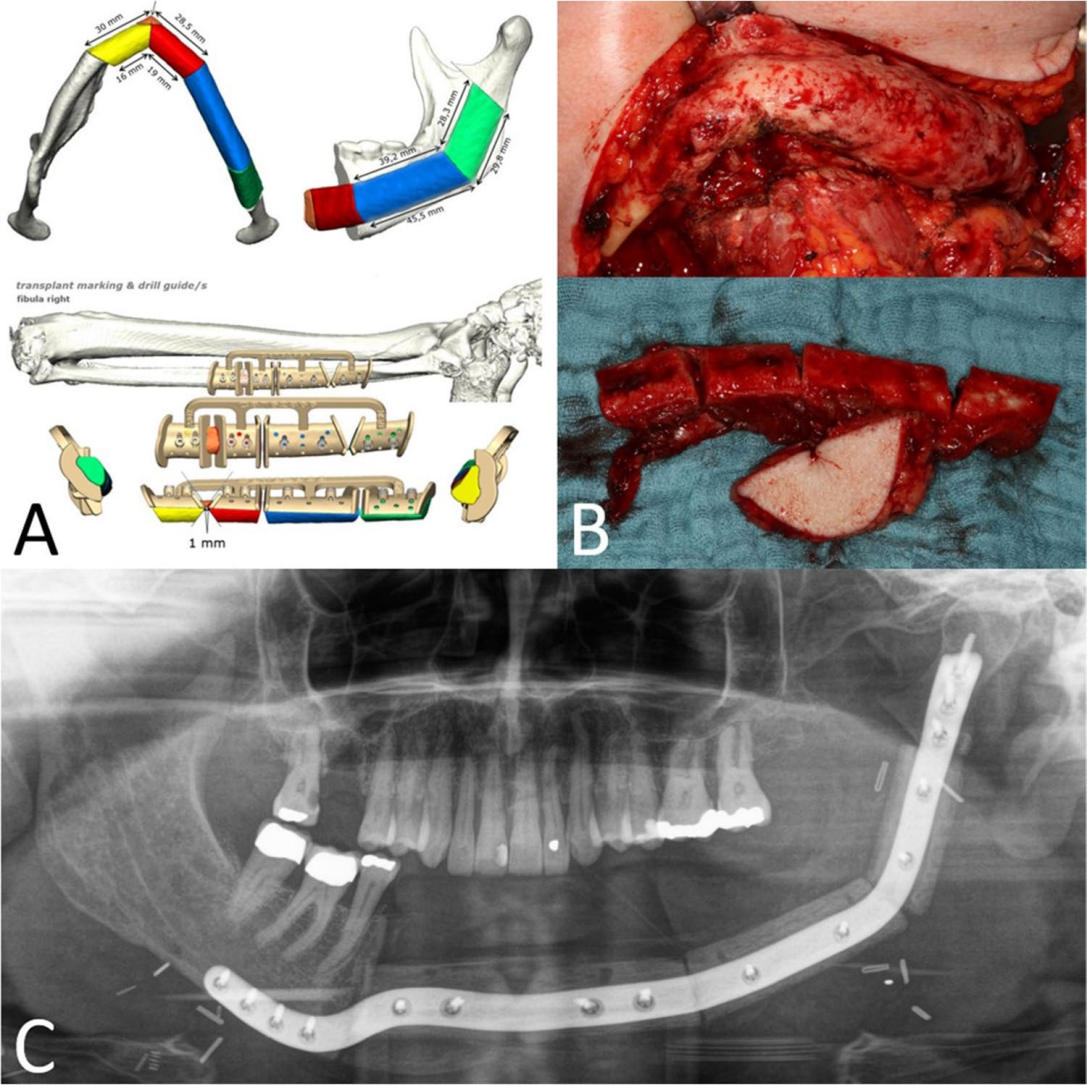
Hemimandibulectomy and reconstruction by free fibula flap. **A** CADCAM-designed fibula graft. **B** Intraoperative situs, fibula graft with 4 segments. **C** Postoperative panoramic radiograph

**Fig. 3:**
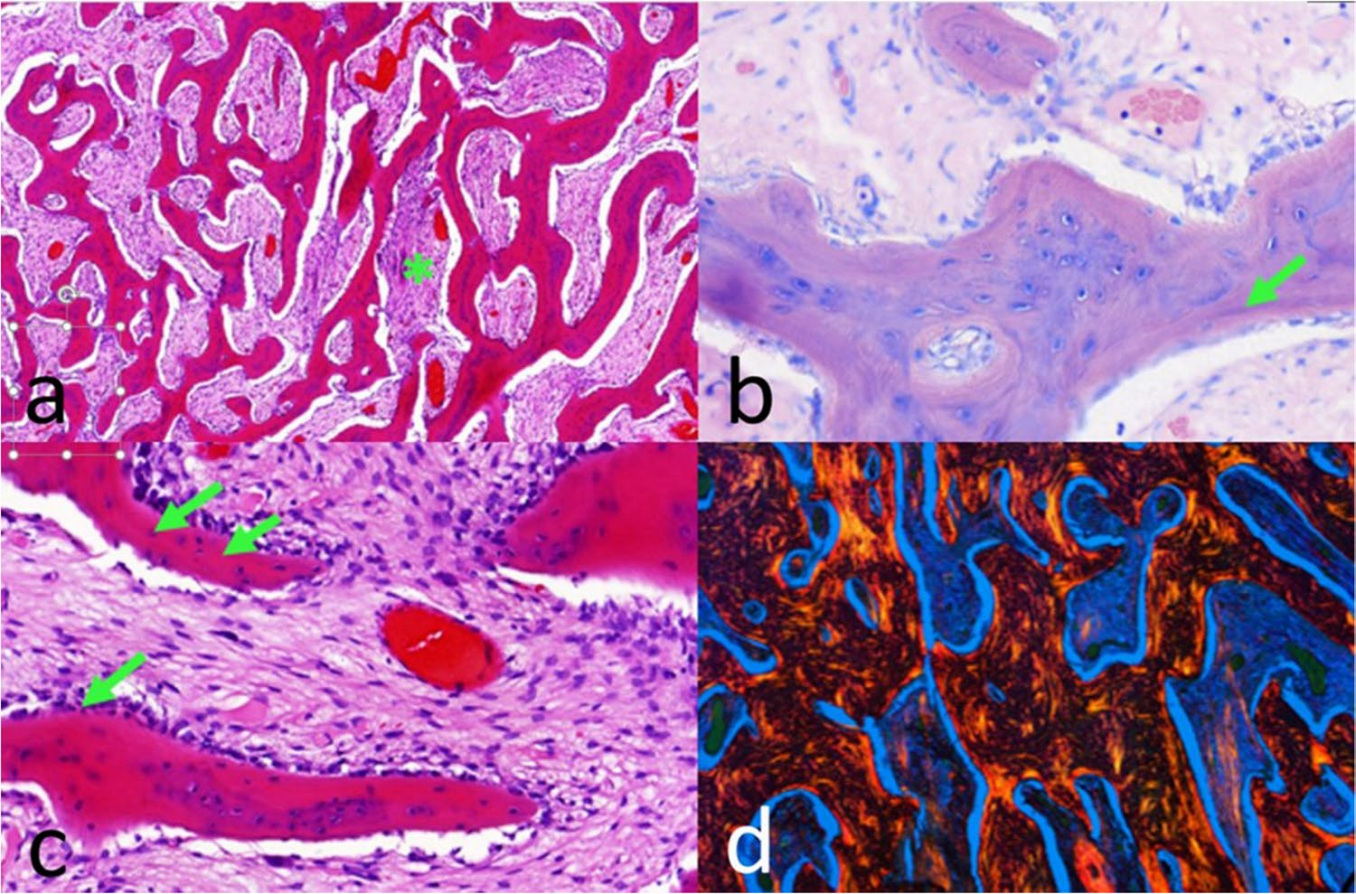
Histological findings. **a** Subperiosteal “new” bone showing reactive woven bone formation with the bony trabeculae arranged almost parallel one to another, note the scant inflammatory infiltrate in the fibrovascular stroma (*) (H.E. stain, × 60). **b** Activation of osteoblasts along of the bony trabeculae (arrow), (H.E. stain, × 250). **c** Many irregular and not parallel bone lines as a result to quick irregular bone production (arrows) (Giemsa stain, × 360). **d** Increased osteoid (trabecular bone surface red–orange) and irregular bone lines (polarized Sirius stain, × 20)

**Fig. 4:**
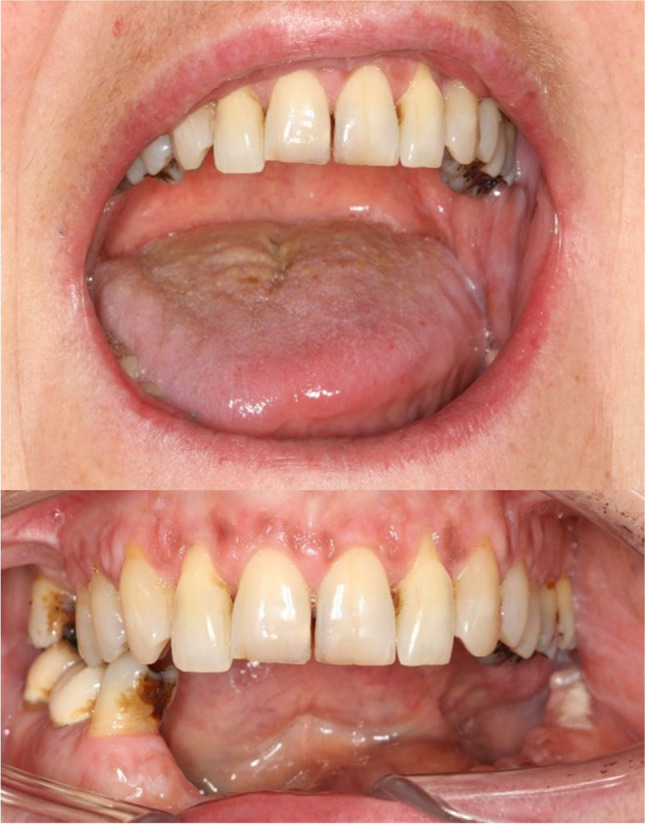
Mouth opening and occlusion 2 months after surgery

In February 2020, a 50-year-old woman presented with a two-year history of intermittent painful swelling, facial asymmetry, cervical fistulation, and bony enlargement of the left mandibular body. She also reported paresthesia of the left inferior alveolar nerve. Repeated oral antibiotic therapy led to a brief ease of symptoms which always returned after stopping antibiotics. Regarding the teeth, there was a decayed left mandibular second premolar which was extracted alio loco. In March 2020, a CT-scan was performed showing a diffused enlargement of the left mandibular body over a distance of 9 cm with a maximal width of 2.1 cm and attenuation of the bone marrow (Fig. 1). Blood count showed a normal total leukocyte number of 8.74/nL and a slightly increased C-reactive protein level of 8.0 mg/L. The left mandibular molars were removed due to advanced periodontitis and a trepan bur biopsy was carried out to harvest a sample of the altered mandibular bone. By histological examination, a chronic inflammation with a fibrosis of the bone marrow and an excess of bone formation could be observed. After discussing the radiologic and histologic results with the patient, the therapeutic decision was made for a subtotal hemimandibulectomy on the left-hand side excluding the condylar process which was singularly not affected by the inflammation. Another reason for keeping the condyle was that 3D planning revealed shortness of the peroneal vessel for contralateral anastomosis when extending the fibula to the condyle region. The resection was performed in June 2020 and the mandible was reconstructed by a CADCAM planned free vascularized fibula graft from the right side (Fig. 2). The fibular skin paddle was placed extraorally for submandibular coverage of the excised fistula and for flap monitoring. The surgical procedure and the postoperative course were uneventful. Histologic assessment of the resected part of the mandible showed a reactive new subperiostal bone formation with numerous irregular bone matrix lines. The new formed bone showed trabeculae arranged almost parallel one to another. Activation of osteoblast zones along the bony trabeculae could be detected as well as fibrovascular stroma within the bony structures with scant chronic inflammatory infiltrate (Fig. 3). On the surface of the new formed trabeculae, an excess of osteoid could be observed altogether compatible with an osteomyelitis associated with a proliferative periostitis [4]. The immediate post-surgical CT scan presented the reconstruction as planned. The patient showed a quick recovery without any complications and was discharged 10 days after surgery. The occlusion was perfect and maximum mouth opening was 3.5 cm (Fig. 4). The patient did not complain any discomfort during follow-up.

**Fig. 5:**
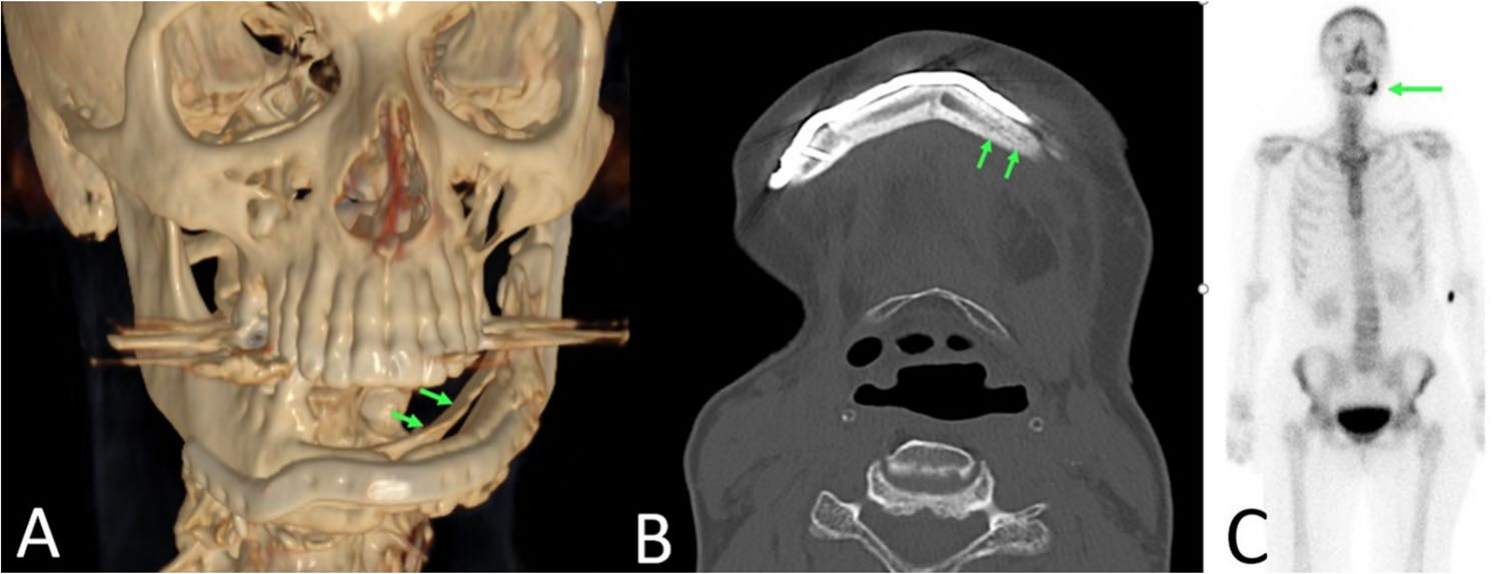
Imaging 6 months after surgery. **A** CT scan in 3D reconstruction: proliferative periostitis manifesting as new bone layer surrounding the fibular transplant (arrows). **B** Axial view showing a widely ossified fibula graft together with moth damage-like osteolysis within the bone (arrows). **C** Bone scintigraphy 6 months after surgery: osteomyelitic relapse in the left mandible (arrow) without any further activation throughout the body (except injection site near left cubital joint)

**Fig. 6:**
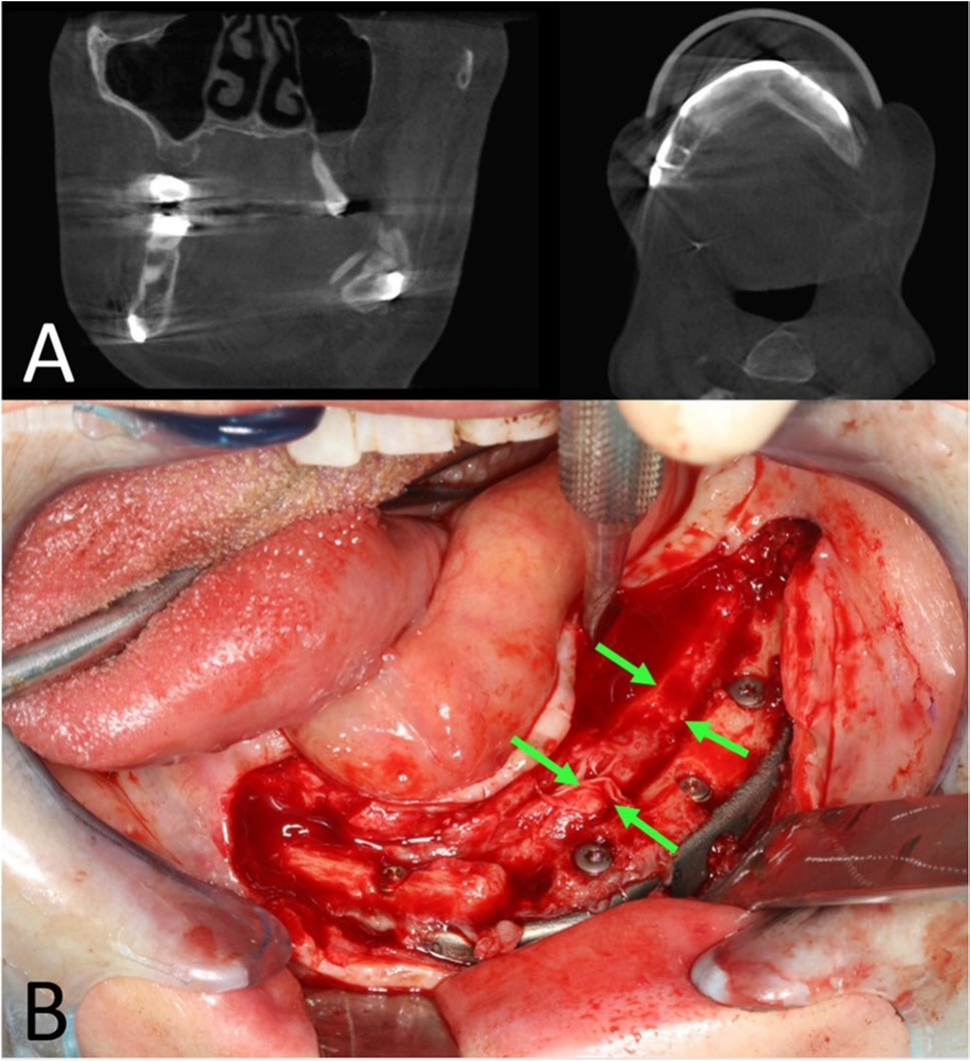
Situation 6 months after ibandronate administration. **A** Cone beam tomography: decrease of moth damage like lesions and stable situation concerning the proliferative periostitis. **B** Intraoperative situation during implant insertion: appositional bone layer surrounding the fibular graft (arrows)

**Fig. 7:**
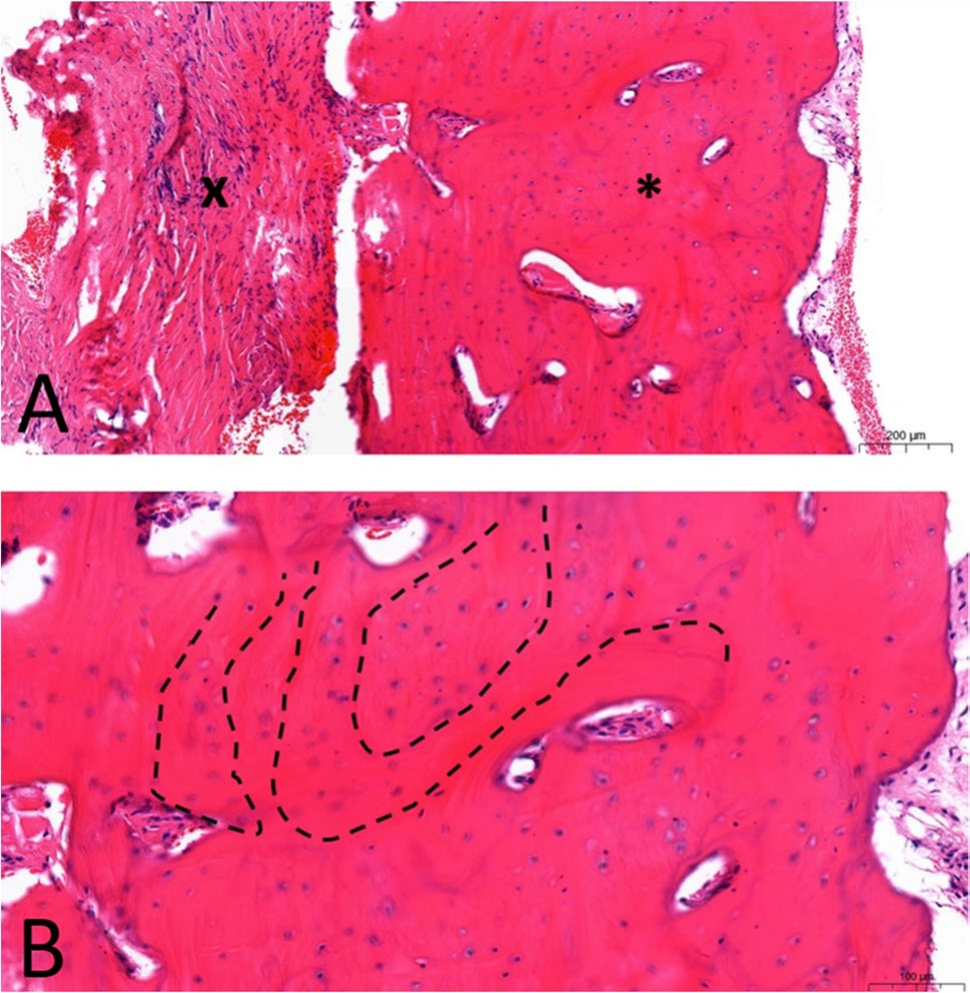
Histological findings after ibandronate application. **A** Fibular transplant: *vital bone, x, subperiosteal fibrosis, note the scant lymphocytic infiltrate as a result of chronic inflammation (stain, H.E.). **B** Lingual bone apposition: vital compact bone with signs of remodeling and irregular, not parallel bone lines (example—- -) (stain, H.E.)

Six months after surgery, she presented with a massive swelling of the reconstructed left mandible. In clinical examination, no fistula could be detected, neither intra- nor extraorally. A panoramic radiograph and another CT-scan showed a new appositional bone formation both lingual and buccal around the fibula transplant further extending to the remaining mandibular bone. The fibula graft itself was widely ossified between the segments and towards the mandibular bone. However, it presented moth damage like lesions pointing to a re-infection caused by a recurrence of the initial osteomyelitis. A SPECT showed inflammatory activation in the left mandibular region (Fig. 5). There were no further activations throughout the body pointing to systemic rheumatic disease. Blood count however only displayed mild leukocytosis (12.5 cells/nL) and a moderate elevation of C-reactive protein (19.5 mg/L). Histological examination of a core biopsy of the retromolar region of the fibular transplant revealed a recurrence of the patient’s known chronic osteomyelitis. Microbiotic investigation did not find any bacteria neither in culture nor in PCR analysis. Ibandronate (Bondronat® 6 mg, Kohlpharma GmbH, Merzig, Germany) as single shot was administered as an off-label trial. Three months afterwards, the patient did not complain any more discomfort. Pain and swelling had markedly declined; no more signs of inflammation could be detected. Leukocyte (7.7 cells/nL) and CRP-levels (0.9 mg/L) were normalized. Cone beam tomography showed a decrease of the moth damage like lesions and a stable situation concerning the periostal new formation of bone (Fig. 6A). In the further course, the patient did not show any more signs of inflammation and stated subjective well-being. After another 3 months, we carried out a partial removal of the osteosynthesis material and the insertion of dental implants (Fig. 6B). As part of the operation biopsies, both of the fibular bone and the periostal apposition were collected. Both samples showed vital bone with a slight fibrosis of the periostal connective tissue (Fig. 7). Nine months after the application of ibandronate, the patient did not complain any more pain, swelling, or other discomfort. Prosthetic rehabilitation was initialized.

## Discussion

There are several terms existing for chronic osteomyelitis with associated periosteal neoformation of bone: Garre’s osteomyelitis, periostitis ossificans, and proliferative periostitis. Formation of subperiosteal bone is discussed to be a reaction of the periosteum to inflammation [3].

At first, the patient presented with a two-year history of recurrent half-sided mandibular swelling and pain. It is possible that the present case started as a common secondary chronic osteomyelitis resulting from an acute infection of the left mandible. CT appearance and panoramic radiograph showed extensive bone marrow reactive changes in addition to the periosteal reactions. This feature combined with not or slightly raised inflammatory markers without positive microbiology culture apart from the patient’s age could also be consistent with the findings in juvenile mandibular chronic osteomyelitis [5]. However, a periosteal reaction with ossification results in bone enlargement in layers, identified as “onion skin” lesions or laminations what is considered to be a characteristic feature in proliferative periostitis [4]. This is concordant with our histologic findings (Fig. 3). Due to the extent of the mandibular destruction, a substantial resection of the mandible in combination with microvascular reconstruction was required.

The unique aspect of this case report is the recurrence of an aggressive mandibular osteomyelitis 6 months after resection and reconstruction with apposition of massive new bone around a moth damage like appearing fibular transplant which itself has completely ossified. There is no comparable case reported in literature. Zhang et al. reported a spontaneous regeneration of bone after the removal of a vascularized fibular transplant from a mandibular segmental defect due to infection [6]. They identified the periosteum as source of osteogenesis and suggested the infection as stimulus for the formation of new bone. The periosteum’s inner layer contains progenitor cells that constantly build and repair bone so it definitely has the capacity for osteogenesis [7]. Additionally, infection may activate osteoblasts originating from the intact periosteum [8]. Other authors report similar cases of the spontaneous bony regeneration between the mandibular stumps after segmental resection [9]. Of course, we did not include the periosteum in resection of the mandible as this is not necessary for benign lesions. Therefore, also in our case, the periosteum is suggested to be the origin of new bone modeling. By contrast to other reports however, the mandible was resected and immediately reconstructed with vascularized bone. After 6 months, the segments displayed regular healing and yet the fibula bone itself presented moth damage like lesions pointing to recurrence of the osteomyelitis affecting the transplanted fibula and to a diagnosis like osteomyelitis with proliferative periostitis which was confirmed by histological examination. Retrospectively, secondary mandibular reconstruction after temporary alloplastic osteosynthesis may have prevented these osteomyelitic changes in the transplanted fibula. However, to the best of our knowledge, this affection of transplanted bone has never been described before. What our case has in common with several other cases of spontaneous bony regeneration of the mandible are the inflammatory conditions because of the patient’s presumably persisting osteomyelitis. It is known that diffuse sclerosing osteomyelitis, condensing osteitis, and proliferative periostitis result in additional bone formation due to a certain focus of infection [10]. So, it is not certain whether the new formation of bone is caused by the remaining mandibular periosteum’s disposition to spontaneous regeneration after a segmental resection or if it is a proliferative periostitis caused by a relapse of the patient’s chronic osteomyelitis. The mean age of patients suffering from proliferative periostitis is around 13 years [11]. The appearance in older patients might be rare but not impossible as we can see in the case of a 69-year-old patient [11]. According to this, a spontaneous regeneration of the mandible after segmental resection is commonly observed in younger patients [8]. Some authors suggest that increasing age might not imply a decrease in periosteal bone-regenerating potential and several cases of spontaneous regeneration of the resected mandible in patients of greater age have been reported [9]. So, our patient’s current age of 51 is not common for proliferative periostitis neither for spontaneous mandibular regeneration but it does not contradict interpreting the new formed bone as both of them. However, the new bone’s shape surrounding the fibula graft, histological examination, the swelling, and the moth damage-like lesions in the fibular bone indicate that it is a periostal reaction in the sense of an osteomyelitic relapse.

In the recent past, some authors suggested the application of bisphosphonates like ibandronate to be successful in reducing clinical symptoms and decreasing disease activity of diffuse sclerosing osteomyelitis [12]. Because of the patient’s osteomyelitic symptoms being resistant to surgical treatment, we started an off-label trial with the application of ibandronate in a single shot dose. This led to a significant symptom decrease. Histological examination of bur biopsies of the fibular transplant and the surrounding bony apposition showed vital bone with a persisting subperiostal fibrosis. We suppose that ibandronate could relieve this osteomyelitic relapse. It is important to emphasize that ibandronate treatment is only described for cases of non-bacterial osteomyelitis such as diffuse sclerosing osteomyelitis [12]. Antiresorptive therapy in patients with a bacterial respectively suppurative osteomyelitis of the jaw may possibly lead to an aggravation of symptoms. In view of the fact that microbiotic investigation was negative in our case, we considered ibandronate to be a therapeutic option.

In conclusion, the presented case of recurrent osteomyelitis appears as a proliferative periostitis despite the patient’s age and although the osteomyelitically destroyed mandibular bone as the infection’s origin had been totally removed which makes this case rather unique.
